# Experimental assessment of cervical ranges of motion and compensatory strategies

**DOI:** 10.1186/s12998-018-0223-x

**Published:** 2019-01-22

**Authors:** Céline Niewiadomski, Rohan-Jean Bianco, Sanae Afquir, Morgane Evin, Pierre-Jean Arnoux

**Affiliations:** 1 Collège d’Ostéopathie de Provence, Aix en Provence, France; 2 iLab Spine - Laboratoire de Biomécanique Appliquée, UMRT24 IFSTTAR - Aix Marseille Université, Faculté de Médecine secteur-Nord, 51 Bd. P. Dramard, 13015 Marseille, France; 3 iLab-Spine - Laboratoire international en imagerie et biomécanique du Rachis, Marseille, France

**Keywords:** Rotation, Cervical motion, Compensatory motion, Motion pattern, Motion strategy, Experimental, Non-invasive motion measurements

## Abstract

**Background:**

Literature is still limited regarding reports of non-invasive assessment of the cervical range of motion in normal subjects. Investigations into compensatory motions, defined as the contribution of an additional direction to the required motion, are also limited.

The objectives of this work were to develop and assess a reliable method for measuring the cervical range of motion in order to investigate motion and compensatory strategies.

**Methods and data collection:**

Ninety-seven no neck-related pain subjects (no severe cervical pathology, 57 women, age: 28.3 ± 7.5y. old, BMI: 22.5 ± 3.2 kg/m^2^) underwent a non-invasive cervical range of motion assessment protocol. In-vivo head’s motion relative to the thorax was assessed through the measurement of the main angular amplitudes in the 3 directions (flexion/extension, axial rotations and lateral inclinations) and associated compensatory motions using an opto-electronic acquisition system.

**Results:**

The principal motion reproducibility resulted in intra-class correlation coefficients ranging from 0.81 to 0.86. The following maximum ranges of motion were found: 127.4 ± 15.1° of flexion/extension, 89.3 ± 12° of lateral inclinations and 146.4 ± 13° of axial rotations after 6 outlier exclusions. Compensatory motions highly depend on the associated principal motion: for flexion/extension: (3.5 ± 7.6;-2.1 ± 7.8°), for rotation: (25.7 ± 17.9°;0.4 ± 4.7)°, for inclination: (22.9 ± 34.7°;-0.04 ± 8.7°). Age, BMI and weight significantly correlated with flexions (*p* < 0.032). Motion patterns were identified through clustering.

**Conclusions:**

This kinematic analysis has been proven to be a reliable diagnostic tool for the cervical range of motion. The non-unicity and variability of motion patterns through the clustering of motion strategy identification have been shown. Compensatory motions contributed to such motion pattern definition despite presenting significant intra-individual variability.

## Introduction

Cervicalgia, perceived as incapacitating for patients, is a medical, social and economic burden on society [1]. This type of pathology has been reported to be associated with advanced age and smoking or exposure to tobacco in childhood [1]. The number of cervicalgia diagnoses is expected to increase as a majority of people are likely to “experience some degree of neck pain in their lifetime” [2]. Neck pain diagnosis is required to accurately manage and address cervicalgia treatment. Indeed, cervical pain in cervicalgia could lead to a modification of the range of motion (ROM), as pain will prevent the subject from fully performing a specific task. The ROM of the cervical spine could then be assessed quantitatively and non-invasively and has been shown to be restricted in patients suffering from cervical radiculopathy and cervicogenic headaches [3].

The cervical segment is a complex articular part of the human body; it can be divided into two parts: the lower cervical part (C3-C7) and the upper one (C0-C2) [4, 5]. According to Watier et al. [6], the spine ROM is defined in each direction of the space and the ROM can be assessed for each functional unit. A wide range of protocols is available to assess the cervical spine ROM. These protocols assess the ROM of the head using: motion acquisition systems [7, 8], inclinometers [9, 10], goniometers [11] or radiography imaging [4]. Main ROM values were provided either by dividing the cervical units as in Frobin et al. [12], or by studying the global ROM. In the no neck-related pain population, the ranges of cervical motion were defined using different protocols. For flexion/extension, a maximum ROM of 87.2° to 145° was found; lateral inclination ranged from 59° to 186°, and rotation from 75° to 175° [6]. In 25 healthy volunteers, flexion was found to equal 68 ± 6.2°, extension 68.3 ± 7.3°, right flexion 49.8 ± 7.5°, left flexion 52.6 ± 7.6°, right rotation 78 ± 6.4° and left rotation 77 ± 7.7° [13]. In 13 healthy volunteers, similar results were found: 79.4 ± 11.7° in extension, 62.2 ± 11.1° in flexion, 46.6 ± 6° in left bending, 48.6 ± 6.9° in right bending, 69.8 ± 7.1° and 71.2 ± 6.4° in left and right rotations [14].

The resolution of the acquisition system and the error associated with data acquisition are one of the issues encountered in the available protocols for cervical ROM measurement. Thus, regarding the measurement of the angular error, very few angular error measurements were reported when others reported 0.1° [15] to 3.92° [16]. Another issue concerns the definition of the reference positioning of the subject before and during the acquisition. Walmsley et al. [17] showed that depending on the subject’s head positioning in neutral position, the ROMs were modified. Such results were confirmed by Wang et al. [18] for flexion extension while contradicted by Sato et al. [19] in a small population.

The complex structure of the spine functional units results in the coupling between large ROMs resulting from combined uni-axial motions. Motions of the adjacent vertebrae are induced by articular surface geometry (different orientation along the spine level) and enable the coupling between axial homo-lateral rotation and lateral inclination motions [20]. At the lower cervical spine level and resulting from the zygapophyseal articulation orientation and from cervical lordosis, lateral inclination could be associated with a homo-lateral rotation and light extension [21]. The intra-individual measurements have shown combined ROM in simple motion, which highlights the variability of motion patterns for a similar instruction or final position [21]. Additionally, compensatory motions are defined as the contribution of an additional direction to the required motion while an alteration of the amplitude of the required motion could be noticed [22, 23]. At the level of the cervical spine structure, several ROM strategies could be used to reach one given objective. Quantitative descriptions of compensatory motions, defined as patterns established unintentionally by the subject in case of cervical pains, are highly limited [24–26] depending on the ROM acquisition technique and the recruitment criteria.

In this context, this work aims 1) to develop a quantitative and reproducible method for the measurement of cervical spine mobility, 2) to measure main and compensatory cervical motions in normal subjects and 3) to characterize motion patterns in this population according to subject characteristics.

## Methods

### Data collection

This study was performed on 101 volunteers according to the inclusion criteria: older than 18 and younger than 65 years old, ability to agree to survey participation, presenting no severe neck pains. Exclusion criteria defined as reported in medical records included severe cervical pathologies, bone fractures or cervical pains, cervico-brachial neuralgia, cardiac or vascular pathologies, pulmonary, cephalic and visceral pathologies, pregnancy or heavy medical treatment. This experimental setting received the approval of the Agence Régionale de Santé for experimentation on healthy volunteers (N°2017–5).

### Experimental layout

**Fig. 1:**
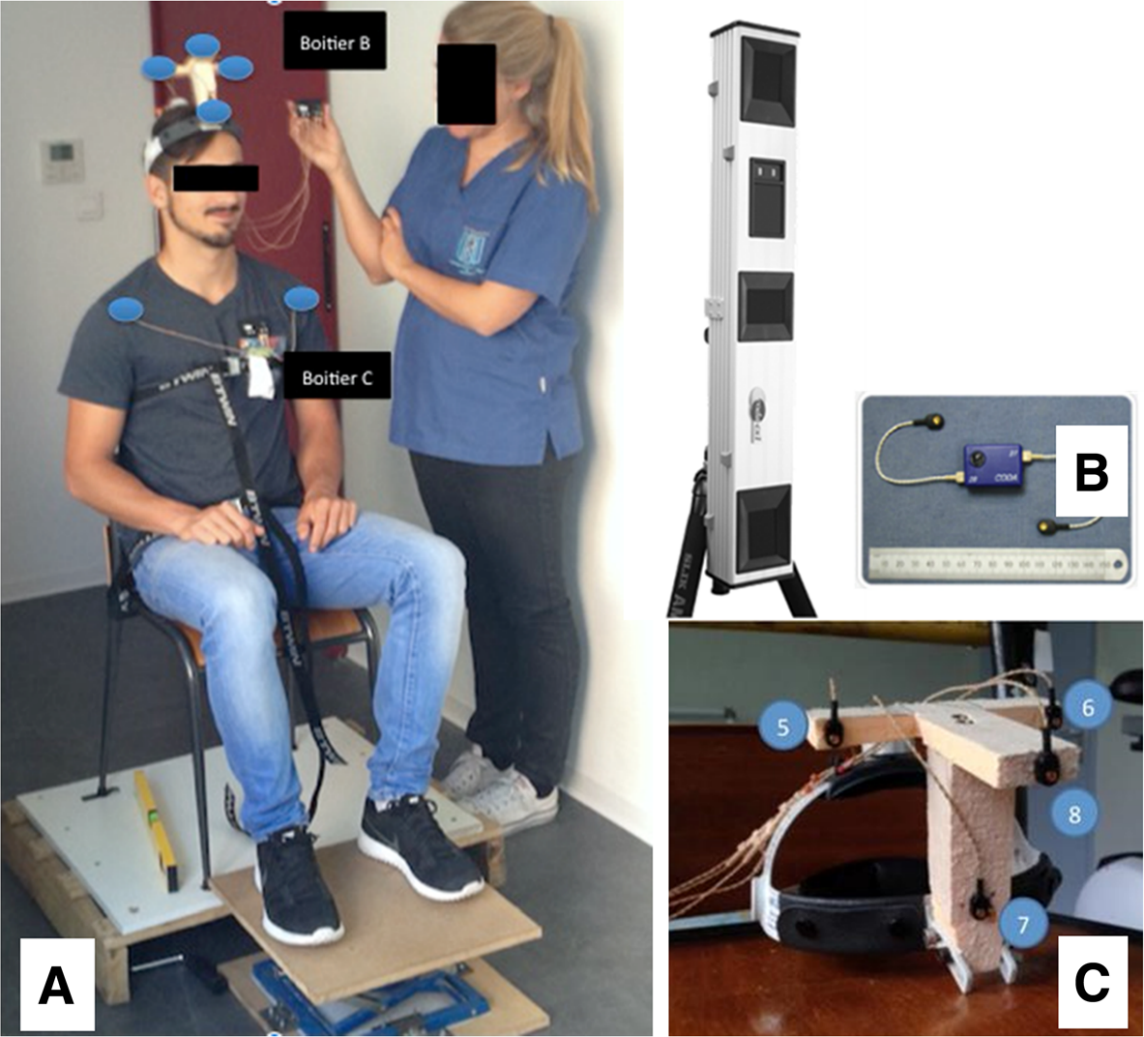
Experimental setting. Positioning of a subject on the experimental chair with thorax ROM reduction straps during a complex motion instruction (**a**). Codamotion (Charnwood Dynamics Ltd.) acquisition system for opto-electronic motion. Aluminium support with 3 video cameras and location sensors (**b**). Headband and opto-electronic sensors (**c**)

Subjects were seated on a specific experimental chair (Fig. 1a). Hip flexion was set to a 90° angular positioning in using the adjustable foot support and a level to ensure horizontality of the thighs. Subjects were positioned on the chair with straps across their pelvis and thorax to avoid improper compensatory motions of the pelvic and scapular waists. The thoraco-lumbar spine was fixed with an adjustable lumbar support to promote physiological lordosis (pelvis set with a slightly retroversion of iliac muscles) and thus create a compensatory physiological dorsal kyphosis that enabled free cervical positioning. The backrest of the chair was in contact with the subject only at lumbar level in order to set the thoracic spine free. Subjects were asked to find a neutral position (horizontal eye trajectory set with a reference on the wall), hands on thighs, palms up to avoid support of the superior limbs and improper movements of the scapular waist. Method repeatability was assessed on 50 subjects selected randomly. These subjects underwent the experimental protocol twice within a fifteen-minute interval.

Subjects were asked to perform several types of motion, starting from a neutral position and returning to this neutral position between each instruction. They were asked to follow their own pace in order to ensure that they stay within their comfort range of motion. Each motion had to be performed with maximum amplitude while staying within the comfort zone (non-forced motion and no pain) and avoiding thoracic motions. The first sequence of motions was composed of 5 flexions followed by 5 extensions and 30 s of rest in the neutral position. The second sequence included 5 axial rotations to the right followed by 5 axial rotations to the left and 30 s of rest in the neutral position. Third sequence was composed of 5 inclinations to the right followed by 5 inclinations to the left and 30 s of rest in the neutral position.

**Fig. 2:**
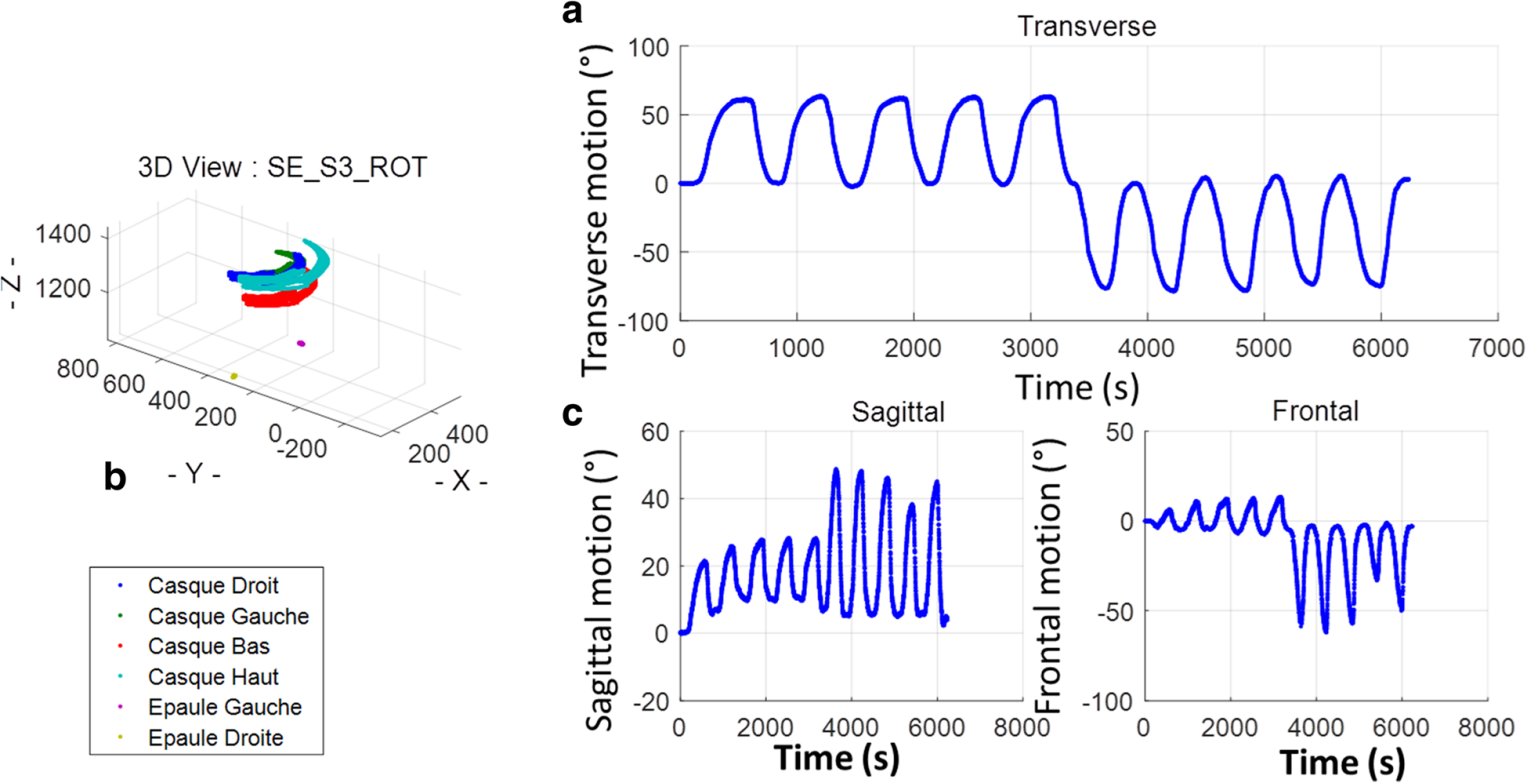
Data analysis representation. Right/left rotations with planar projections (transvers. Sagittal and frontal) and 3D representation. Subject SE-S3: 24 years old, male. Compensatory motion. Positioning of a subject on experimental chair with thorax ROM reduction straps during a complex motion instruction (**a**). Right/left rotations with planar projections (transvers, sagittal and frontal) and (**b**) 3D representation. Subject SE-S3: 24 years ols, male. (**c**) Compensatory motions

The motion acquisition system used to capture motions of the subjects was a Codamotion (Codamotion CX1, Charnwood Dynamics Ltd., UK) system. It is composed of 3 pre-calibrated video cameras in an aluminium support and of active opto-electronic markers (4 sensors per sensor box) located in front of the subject’s seat and enabling maximum visibility of the markers (Fig. 2-ab). Marker displacements were tracked in the 3 directions of the space, using specific infrared frequencies. Two sensors were placed on the shoulders of the subject at the coracoid apophysis level. A rigid adjustable headband instrumented with 4 sensors (fixed in a T shape on the anterior part of the head band) was placed on the subject’s head (Fig. 1-c). Acquisition was performed at a frequency of 100 Hz and spatial resolutions of 0.1 mm. Measurements were performed with the ODIN software (Charnwood Dynamics Ltd.). The acquisition system was aligned to define the origin at the centre of the chair: Z axis defined as the caudo-cranial direction, X axis as the medio-lateral direction on the subject’s left and Y axis in the antero-posterior direction. The relative locations of the 4 sensors on the headband were fixed during the experiment. Any missing data caused by the obstruction of sensor visualisation by the cameras was computed with a specific algorithm using the 3 known sensor locations to deduce the fourth one.

### Kinematic data analysis

Exported data was post processed using custom-made Matlab (The MathWorks, Inc.) scripts for this study.

For each motion, neutral initial positioning was used as a reference. The angular variations of the 4 sensor positions were measured in each plane projection (XY, XZ, YZ) and in the neutral position. The angular variations of the sensors for the flexion-extension motions (sagittal plan), axial rotation (transverse plan) and inclination (frontal), were measured for each acquired time step. For each motion, maximal amplitudes as well as standard deviations on 5 similar motions were computed to avoid false motions or effects related to subject weariness (Fig. 2).

Neutral positions were quantified for each motion sequence (flexion/extension, inclination and rotation) to identify the variability of the initial position between two consecutive motions. Compensatory motions were defined as maximum amplitude on the two other planes at the same time as the associated principal motions: for flexion/extension motion, transverse and frontal compensatory motions were computed, for right and left rotations, sagittal and frontal compensatory motions and for right and left inclination, sagittal and transverse compensatory motions.

### Statistical methods

First, univariate statistical analysis was performed to insure data homogeneity. Principal component analysis (PCA) was performed on the principal motions analysis and used to identify outliers, taking into consideration the first five axes (presenting 80% of inertia). Method repeatability was tested on 50 subjects on account of time constraints and sufficient in terms of statistical power for such quantification. It was assessed using intra-class correlation coefficient (ICC – consistency ICC for one-way random effects model) for each principal motion. Clusters of the 97 subject population studied (without outliers) were identified according to the coordinates of the first five axes of the PCA of principal and reliable compensatory motions (ICC > 0.75) using agglomerative hierarchical clustering (AHC- Warel method). Additionally, ANOVA for the assessment of cluster group influence and linear regressions between morphological characteristics (gender, age, height and weight) and global ROM measurements (flexion + extension, right/left rotation and right/left inclination) were tested. Statistical analyses were performed using R software. Significant results were considered with a *p* value < 0.05.

## Results

### Method reliability

The neutral position as selected as the minimum angular value between two consecutive motions was found to equal 3.0 ± 2.5°, with an intra-individual variability within the same motion of 6.8 ± 3.8° on average in flexion/extension, respectively 1.6 ± 2.5° and 5.3 ± 2.7° in inclination and 2.2 ± 2.2° and 4.2 ± 2.1° in rotation (Table 1).

**Table 1:** Maximum amplitude of ROM principal motions. Intra-individual variability of the neutral position. Repeatability of the ROM measurement in 50 no neck-related pain volunteers. Annotation: values are presented as mean (standard deviation)

	Total	Neutral position	ICC (95%)
Gender (Female)	101 (57)		
Age (y.)	28.3 (7.5)		
Weight (kg)	66.2 (12.4)		
Height (cm)	171 (8.8)		
BMI (kg/m2)	22.5 (3.2)		
Flexion (°)	65.3 (12.2)	3 (2.5)	0.81 (0.68–0.88)
Extension (°)	−60.9 (9.2)	6.8 (3.8)	0.81 (0.69–0.89)
Right Rot. (°)	71.2 (8.1)	2.2 (2.2)	0.82 (0.7–0.89)
Left Rot. (°)	− 74.5 (8.1)	4.2 (2.1)	0.83 (0.72–0.9)
Right Incl. (°)	43.3 (12.2)	5.3 (2.7)	0.92 (0.86–0.95)
Left Incl. (°)	−45.3 (9.2)	1.6 (2.5)	0.80 (0.67–0.88)

Reproducibility between the test and retest on 50 subjects of the method was assessed using an intra-class coefficient. This resulted in an ICC of 0.81 for flexions/extensions, of 0.86 for right/left inclinations and of 0.83 for right/left rotations. Regarding compensatory motions, the ICC averaged 0.41 and 0.26 for flexions/extensions in transverse and frontal planes respectively, 0.81 and 0.75 for sagittal and transverse planes in inclinations and 0.89 and 0.38 for sagittal and frontal planes in rotations.

The missing data, which had to be computed by the dedicated algorithm using the 3 known sensor locations, was 9.7% on average.

### Ranges of motion

The cohort included 42 males and 55 females (age: 28.3 years range 21–53*,* median: 25 years; weight: 66.4 kg range 41 to 106 kg; height: 171.3 cm, range 152 to 196 cm). Ranges of principal motions are presented in Table 1 for the entire population without outliers. Eighty subjects practiced a physical activity on a regular basis (no professional practice) and 49 had suffered cervical pain (related to car accidents or sports) (simple whiplash, physiological curve inversion, cervical sprain, associated cervicalgia). Maximum flexion/extension angular motions were found of 126.8 (15.7)°, maximum right and left inclinations equalled 88.8(12.4)° and axial rotations equalled 146.1(13.3)° (Table 3). Intra-individual standard deviation was found 2.4(1.7)° for flexion/extension, 1.7(1.3)° for rotations and 1.4(1.7)° for inclinations.

### Cluster analysis and motion patterns

Analysis of the ascending hierarchy classification of the population enabled the definition of 3 clusters. The first cluster included 30 subjects more advanced in age, with a similar gender ratio to the entire population, a higher BMI, a lower average ROM compared to the entire population, and the highest reduction in flexion/extension (− 12.3% on average for both) and inclinations (− 11.2%, rotations: − 10%) (Table 2).

**Table 2:** Ranges of principal motion of painless ROM within the 3D space in 97 subjects. Reference values for cluster populations. Annotation: values are presented as mean (standard deviation)

	Cluster 1	Cluster 2	Cluster 3	Total	p
Population (Female)	30 (16)	32 (15)	35 (24)	97 (55)	
Age (y.)	31.8 ± 9.7	27.7 ± 6.8	26 ± 4.8	28.3 ± 7.5	0.002
Weight (kg)	69.9 ± 15.2	66.8 ± 11.1	63 ± 10.5	66.4 ± 12.5	0.2
Height (cm)	171.2 ± 9.5	172.8 ± 8	169.9 ± 8.7	171.3 ± 8.7	ns
BMI (kg/m2)	23.7 ± 4.1	22.3 ± 2.7	21.7 ± 2.6	22.5 ± 3.2	0.1
Flexion (°)	55.3 ± 9	71.2 ± 8.6	69.9 ± 10.1	65.8 ± 11.6	< 0.001
Extension (°)	−55.7 ± 9.1	− 63.3 ± 8.8	−63.3 ± 7.5	− 61 ± 9.1	< 0.001
Right Rot. (°)	64.6 ± 7.3	75.6 ± 5.2	74.1 ± 6	71.7 ± 7.8	< 0.002
Left Rot. (°)	−66.9 ± 6.7	−78.3 ± 6.4	−77.6 ± 4.4	−74.5 ± 7.8	< 0.003
Right Incl. (°)	38.4 ± 6.2	49.6 ± 6.4	42 ± 5.2	43.4 ± 7.5	ns
Left Incl. (°)	−40.4 ± 6.2	−51.4 ± 5.2	−44.2 ± 4.5	−45.4 ± 6.9	ns
Mean Time Flex/Ext	362 ± 89.2	391.1 ± 68.1	393.6 ± 83.4	383 ± 81	ns
Mean Time Rot	287.4 ± 64.4	314.4 ± 65.4	305.9 ± 76.5	303 ± 69.5	ns
Mean Time Incl.	282.9 ± 65.4	295.9 ± 62.1	296.7 ± 77.5	292.2 ± 68.6	ns

In the second cluster, presenting an equal number of male subjects, slightly heavier and with a similar BMI, the ranges of motion were found to be higher than in the entire population (+ 6% for flexion/extension, + 13.7% for inclinations and 5.3% for rotations). The third cluster of 35 subjects presented a similar average ROM to the entire population, more women, younger and with a lower BMI. Significant differences between clusters were found for all range of motion measurements (AOV: *p* < 0.003) except for inclinations.

Averaged times required to perform each task are reported in Table 2. Times for flexion/extension motions were found to have required slightly longer (383 ± 81 ms) than rotations (303 ± 70 ms) and inclinations (292 ± 69 ms). Average times did not differ significantly between clusters.

### Compensatory motions analysis

Compensatory analysis presented high inter-individual variability and values depending on the associated principal motions (Table 3). For flexion/extension, the amplitudes of compensatory motions were similar between transverse and frontal planes (maximum 11°, − 0.4 ± 4.7 and 3.4 ± 7.6 in the entire population), while main compensatory motions were found in sagittal planes for both rotation (maximum 33.7, 22.7 ± 33.9 in the entire population vs. frontal: vs. maximum 1.8, − 2 ± 8 in the entire population) and inclination (transverse: maximum 36.8, 25.7 ± 20.6 in the entire population vs. sagittal: maximum − 1.9, 0.4 ± 9.1 in the entire population).

**Table 3:** Compensatory motion amplitudes and principal motions in 97 subjects. Reference values for cluster populations. Annotation: values are presented as mean (standard deviation)

	Cluster 1	Cluster 2	Cluster 3	Total	p
Population (Female)	30 (16)	32 (15)	35 (24)	97 (55)	
Flexion/extension (°)	111 ± 13.6	134.5 ± 8.4	133.2 ± 12.6	126.8 ± 15.7	< 0.001
Rotation (°)	131.4 ± 10.9	153.8 ± 8.1	151.7 ± 7.9	146.1 ± 13.3	< 0.001
Inclination (°)	78.9 ± 9.3	101 ± 9.3	86.2 ± 7	88.8 ± 12.4	0.04
Trans Flexion/Extension (°)	−0.7 ± 4.7	0.8 ± 5.2	0.9 ± 4.3	0.4 ± 4.7	ns
Front Flexion/Extension (°)	5 ± 8.1	1.5 ± 8.2	3.9 ± 6.2	3.4 ± 7.6	ns
Sag. Right/Left Rot. (°)	13.8 ± 15.4	28.6 ± 38.6	24.9 ± 39.8	22.7 ± 33.9	ns
Front. Right/Left Rot. (°)	2.6 ± 6	−0.9 ± 7.4	−6.9 ± 7.4	−2 ± 8	< 0.001
Sag. Right/Left Incl. (°)	0 ± 8.6	5.4 ± 9.1	−3.7 ± 7.4	0.4 ± 9.1	0.07
Trans. Right/Left Incl. (°)	17.9 ± 15.5	26.2 ± 26	31.9 ± 16.7	25.7 ± 20.6	0.006

### Correlations with subject characteristics

Correlation analysis between subject characteristics (gender, weight, height, BMI and age) and principal ROMs showed significant a correlation between weight and flexion (*r* = − 0.19, *p* = 0.03). BMI significantly correlated with flexion (*r* = − 0.29, *p* = 0.003). Age correlates with all principal motions (*p* > 0.15, Table 4).

**Table 4:** Linear regression between subject characteristics and principal motions: adjusted R value (*p* value): *** depicted *p* < 0.001. ** depicted *p* < 0.01 and * depicted *p* > 0.05

r(p)	Gender (M/F)	Weight (kg)	Height (cm)	BMI (kg/m2)	Age (years)
Flexion/extension	0.07 (0.24)	−0.19 (0.032)*	0.1 (0.81)	−0.29 (0.003)**	− 0.35(> 0.001)***
Rotation	0.09 (0.45)	−0.07 (0.23)	0.1 (0.96)	−0.13 (0.102)	− 0.26 (0.006)**
Inclination	−0.05 (0.14)	0.09 (0.18)	0.03 (0.35)	0.06 (0.247)	−0.23 (0.015)*

## Discussion

### Reliability of the methods

Results on the reliability of the principal motion measurement method, assessed during a test and retest at a fifteen-minute interval, showed good reproducibility (ICC ≥80). The reproducibility of a subject’s response to an instruction will be influenced by the reproducibility of the neutral position which has been assessed here. It was shown to be lower than 6.8° maximum in extension (ranging between 1.6 and 5.3 for others motions). Additionally, intra-individual standard deviation was limited (found maximum in flexion/extension at 2.4° on average) and far under the neutral position showing higher reproducibility of maximum amplitude value. The reproducibility of compensatory motion analysis was irregular depending on the associated principal motion. Compensatory motions associated with inclinations and those within the sagittal plane associated with the rotation motions could be considered as reproducible (ICC > 0.75) while others should be used carefully. Such results could be explained by motion mechanisms (inclination is more likely to induce a higher compensatory motion than flexion/extension; similarly, rotation is more likely to induce a compensatory motion in the sagittal plane).

### ROM results

The angular values of the main motions in this cohort were similar to the ones reported in the literature by several authors [6]: flexion and extension: 87.2° to 145° (our result on average: 126.8°), lateral inclination on average: 59° to 186° (our result: 88.8°) and rotation 75° to 175° (our result on average: 146.1°). Depending on the range of age, weight, height and gender of the participants, results vary from one study to another. When similar age ranges to our cohort were considered, similar results were found [27].

The average time required to perform the task was not found to differ between clusters in our result, however the differences in terms of age and BMI, while significant, remained limited. Such differences may not be substantial enough to show changes in terms of average time previously reported in the literature [28].

### Compensatory motions analysis

As previously highlighted in the literature [20, 21], coupling between cervical spine motions has been shown in the lateral inclination motion. Coupling between homo-lateral rotations was highlighted in the lower cervical vertebrae (C3-C7), taking into account sub-occipital and cranial rotations in opposite direction to the inclination [29]. Such compensatory motions were reported to show a functional outcome of the head and spine and were explained by the anatomy of the inclination and articular facet. Quantifications of such compensatory motions were limited [30] and our results show the feasibility of such quantifications and their inputs (cluster analysis) in the cervical motion analysis despite the high inter-individual variability. Such inter-individual variability could then be explained by both lifestyle and low significance dysfunction.

High values were found in the sagittal planes for rotations and inclinations. For right and left axial rotations, all subjects presented large variations in the sagittal plane. For inclinations, compensatory motions in the sagittal plane were also found to be necessary in order to perform the required motion. Flexion/extension presented the more balanced measurement in terms of compensatory motions.

### Cluster analysis

To perform the required instruction, the subject will use different cervical mobility patterns (principal motions and compensatory motions). This cluster analysis revealed significant differences in the compensatory ROM, while principal motion differences were found to differ significantly between clusters. This highlights the non-unique motion pattern to reach a similar displacement. Thus, frontal plane contributions to principal rotation and transverse plane contributions to inclination motions were highlighted. Several results could explain differing motion patterns within the population: anatomical variability (skeletal), muscle strength and activation during the task, as well as physical activity and lifestyle. While the time required to perform a task has been proven to influence motion patterns [31], no significant differences in average times were reported in our results.

### Correlation with subject characteristics

The correlation analysis showed that all range of motion will reduce with age, which was already highlighted previously in the literature [30, 32]. The effects of aging tested here are limited as far as the correlation coefficient between motions and age are concerned, probably due to the limited number of available volunteers. However, as highlighted in the first section of the discussion, our results were found to be similar to the literature whenever the age of the cohort was similar [13, 14]. This could suggest that our cohort age would require to be widened in order to validate the correlation between age and principal motion. Such influence has been explained in the literature through the physiological degenerative mechanism of the spine increasing disk and posterior articulation stiffness, decreasing ROM on the transverse and sagittal planes [33, 34]. Such interpretation highlights the main role of intervertebral disks in axial rotation and flexion/extension as well as the significance of their alterations.

Additionally, the slight correlation between weight and flexion could be explained by the increase in the muscular and adipose mass of the visceral area and of the neck. Similar results were found in Malmström et al. [30] regarding weight, height and age while showing that older, overweight subjects had decreased overall ROM. In contrast, slightly younger females with lower weight compared to the group average had higher or similar ROM (cluster analysis: cluster 1 vs. cluster 3).

### Limitations

Experimental acquisition systems as well as protocol and data analysis have limitations, which partially explain the variability of the measurements found in the literature. In this study, error related to protocol was intended to be limited by initial subject positioning (head and thorax postures) as well as by the codaMotion acquisition method, which enabled initialisation of the subject posture to a reference similar in all subjects while remaining specific to each subject. Despite these efforts, by increasing the number of sensors on the cranium, face, sternum and clavicles, we would improve the reliability and precision of the results and would be adding data to characterize ROM. Inter-days and inter-operator reproducibility for marker positioning should also have been tested. Similarly, a second acquisition system could help in measuring front and back motions. Additional data analysis, including helicoidally defined reference system analysis, has been developed but could however not be tested with the present set of data [35]. Medical questionnaires or indexes could have been used to further describe the selected population as in [25]. Symmetrical analysis could also be performed, requiring the laterality of the subjects. Finally, despite the limited number of volunteers we were able to recruit, results show a correlation between ranges of motion, BMI and weight. A larger cohort could however be considered to further confirm these results and enable a closer investigation into the correlation with age. Further analysis is needed to fully assess the benefits of compensatory motion analysis regarding cervicalgia treatment.

## Conclusion

This work has provided description and testing of a robust and reliable protocol in order to measure main cervical ROM through head to torso motion detection. Compensatory motion measurements as well as the reliability of the method have been quantified and the principal motion amplitude could thus be defined as a normal reference value for cervical ROM in the specific age range of our cohort. Cluster analysis highlighted the need to assess compensatory motions and revealed the non-unicity of the motion pattern when following a similar instruction in no neck-related pain volunteers. Additionally, age, BMI and weight were shown to correlate with mostly flexion-related ranges of motion. Further work is needed to understand how such quantitative methods could objectively contribute to diagnostic and treatment.
